# Using n-gram analysis to cluster heartbeat signals

**DOI:** 10.1186/1472-6947-12-64

**Published:** 2012-07-08

**Authors:** Yu-Chen Huang, Hanjun Lin, Yeh-Liang Hsu, Jun-Lin Lin

**Affiliations:** 1 Department of Mechanical Engineering, Yuan Ze University, Taoyuan, Taiwan; 2 Department of Information Management, Yuan Ze University, Taoyuan, Taiwan; 3 Gerontechnology Research Center, Yuan Ze University, Taoyuan, Taiwan

## Abstract

**Background:**

Biological signals may carry specific characteristics that reflect basic dynamics of the body. In particular, heart beat signals carry specific signatures that are related to human physiologic mechanisms. In recent years, many researchers have shown that representations which used non-linear symbolic sequences can often reveal much hidden dynamic information. This kind of symbolization proved to be useful for predicting life-threatening cardiac diseases.

**Methods:**

This paper presents an improved method called the “Adaptive Interbeat Interval Analysis (AIIA) method”. The AIIA method uses the Simple K-Means algorithm for symbolization, which offers a new way to represent subtle variations between two interbeat intervals without human intervention. After symbolization, it uses the n-gram algorithm to generate different kinds of symbolic sequences. Each symbolic sequence stands for a variation phase. Finally, the symbolic sequences are categorized by classic classifiers.

**Results:**

In the experiments presented in this paper, AIIA method achieved 91% (3-gram, 26 clusters) accuracy in successfully classifying between the patients with Atrial Fibrillation (AF), Congestive Heart Failure (CHF) and healthy people. It also achieved 87% (3-gram, 26 clusters) accuracy in classifying the patients with apnea.

**Conclusions:**

The two experiments presented in this paper demonstrate that AIIA method can categorize different heart diseases. Both experiments acquired the best category results when using the Bayesian Network. For future work, the concept of the AIIA method can be extended to the categorization of other physiological signals. More features can be added to improve the accuracy.

## Background

Biological signals may carry specific characteristics that reflect basic dynamics of the body. In many studies, biological signals are mapped into symbolic sequences for further analysis. For example, the DNA-sequence, which is composed of adenine (A), cytosine (C), guanine (G) and thymine (T), is a well-known biological symbolic sequence. When mapping to symbolic sequences, the essential information of the original signals must be preserved.

The human heart beat time series is another well-studied example. Human cardiac autonomic activity is affected by two different interactions: sympathetic activity increases heart rate, and parasympathetic activity decreases heart rate. Since these opposite effects are stimulated by many different kinds of stimuli, human heart beat time series is highly variable and complex. [Cysarz et al. 1] demonstrated that even regular heartbeat dynamics may be associated with cardiac health. They found that in healthy subjects, continuous adaptation to different activities occurs during daytime, but there was erratic behavior in Congestive Heart Failure (CHF) patients.

Regular heart beat dynamics contains distinct alternation of acceleration and deceleration. Some early traditional linear methods could reliably describe partial actions in autonomic regulation, such as respiration [2,3]. However, non-linear methods are needed to analyze highly variable data, such as heartbeat signals [2,4,5]. In recent years, many researchers have shown that representations which used non-linear symbolic sequences can often reveal much hidden dynamic information. This kind of symbolization proved to be useful for predicting life-threatening cardiac diseases [6-11].

At present, there are three different approaches for using non-linear symbolic sequences to represent heart beat time series. The first approach is based on the deviation of the heart rate time series from the local mean, and a symbol is assigned to each heartbeat. For example, if the momentary heart rate is close to the mean value, it is assigned a “1”; if the heart rate is lower than the mean value, it is assigned a “2”; others are assigned a “3”. [Voss et al. 7] found that there were some specific patterns in patients after suffering myocardial infarction using the symbolization based on deviation from the mean value. They later improved this method to identify patients with other high risk cardiac diseases [12].

The second approach is to symbolize the increase or decrease of the momentary heart rate by two different symbols. For example, Yang et al. [10] simplified the heartbeat dynamics via mapping the output to binary sequences, where the increases of the interbeat intervals were denoted by “1” and others were denoted by “0”. They presented a distance method based on rank order statistics to calculate the dissimilarity between two symbolic sequences. According to the results, this method can robustly recognize the difference between healthy people and patients with heart diseases. [Peng et al. 11] of the same research team, combined the distance method with a weighting function, resulting in less overlap between groups, and more clearly distinguished classes corresponding to the level of subjects in the CHF group. [Van et al. 13] also found that symbolization can be applied to quantify the fetal heart rate, demonstrating that development of the autonomic nervous system and emergence of behavioral states lead to increase in both irregular and regular heart rate patterns.

The third approach is to divide the range between minimum and maximum heart rate into a few equidistant intervals, or to map a time series onto a symbolic sequences of permutation rank [14-16]. Entropy and entropy rate were used to evaluate the complexity of heart variability. [Porta et al. 14] used the pattern classification method to auto identify different physiological conditions by the activation of different mechanisms responsible for cardiovascular regulation. Permutation entropy and modified permutation entropy analysis have also been studied, which maps a time series onto a symbolic sequence of permutation rank [15,16].

The second approach described above for symbolization does not need any parameter settings (e.g., the mean heart rate is required in the first approach), and it is independent of any other features of heart rate variations. In contrast to the third approach described above, it does not need to adjust the range of intervals which might affect the results of classification. However, the second approach used only binary symbols (e.g., 0 and 1) to represent acceleration and deceleration of interbeat intervals, which might not be able to represent the degree of variations. For example, the difference between two interbeat intervals such as +250 and +100 may both be represented as acceleration and assigned “1”, but actually they are not the same in a detailed interpretation, and the degree information of acceleration is lost in this binary representation.

To address this problem, this paper presents an improved method called the “Adaptive Interbeat Interval Analysis (AIIA) method”. The AIIA method uses the Simple K-Means algorithm for symbolization, which offers a new way to represent subtle variations between two interbeat intervals without human intervention. After symbolization, it uses the n-gram algorithm to generate different kinds of symbolic sequences. Each symbolic sequence stands for a variation phase. Finally, the symbolic sequences are categorized by classic classifiers.

This paper is organized as follows. Section 2 describes the procedure of the AIIA method. Sections 3 and 4 present two experiments to validate this method in classifying different diseases. Finally, Section 5 concludes the paper.

## Methods

**Figure 1:**
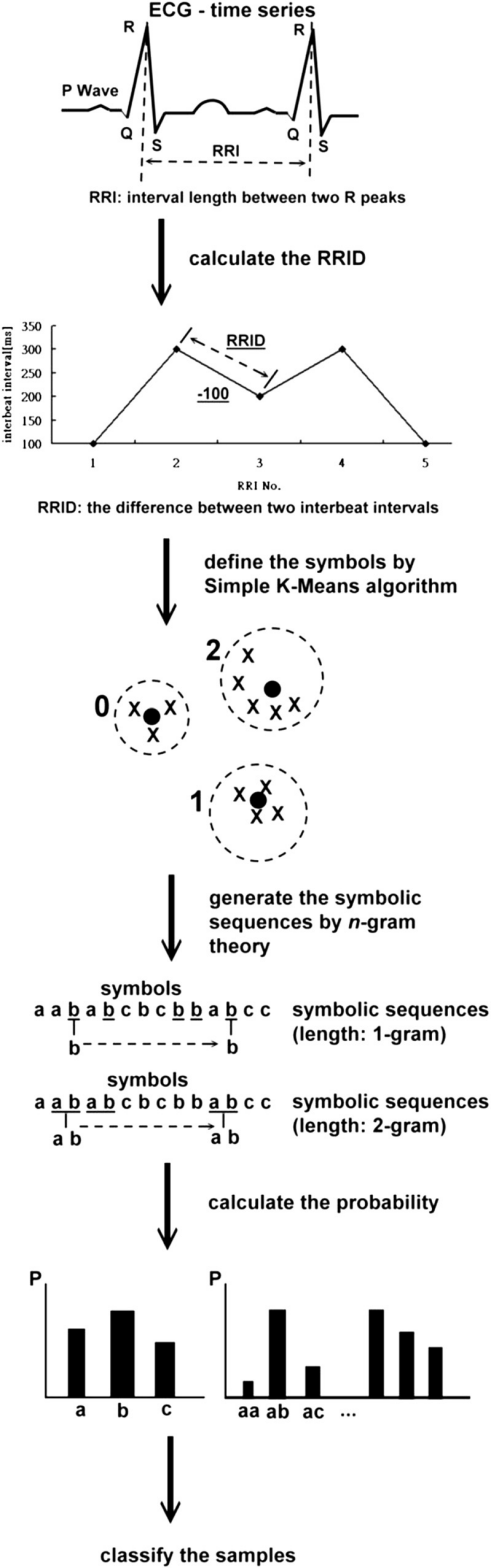
Concept flow chart of the AIIA method.

Figure 1 is the concept flow chart of the AIIA method. First, the Inter-beat (RR) intervals (RRI) from the ECG time series are extracted and the RRI differences (RRID) of each sample are calculated. Then the RRI differences are symbolized using the Simple K-Means algorithm. Styles and signatures are then identified using the n-gram algorithm. Finally, the probability of each signature is calculated as the input to the classic classifiers. Details of the 5 steps are described as follows.

### Preliminary treatment – Calculating the RRI difference

**Figure 2:**
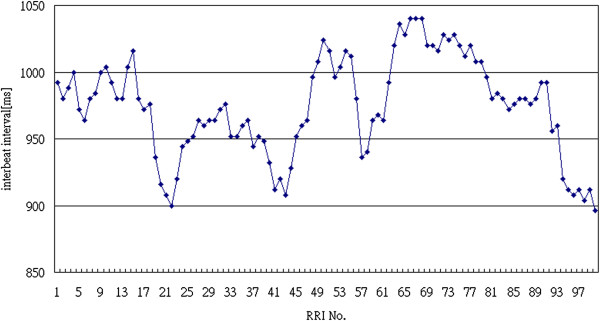
Typical example of interbeat interval time series.

**Figure 3:**
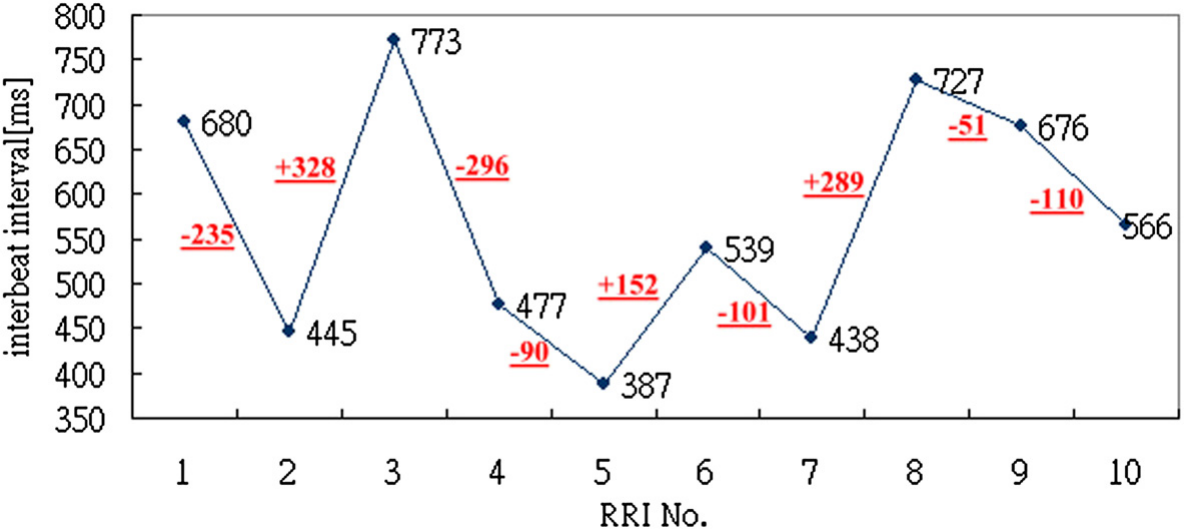
Values of RR intervals and RRI differences.

Figure 2 is a typical example of an interbeat interval time series. Consider an interbeat interval time series where *x*_*i*_ is the *i*-th interbeat interval. RRI difference (*RRID*_*i*-1_) is the difference between *x*_*i*_ and *x*_*i-*1_. Calculating each pair of successive interbeat intervals, Figure 3 demonstrates the RR intervals and the RRI differences.

### Symbolization – Using Simple K-Means algorithm to cluster the RRI differences

Simple K-Means is one of the most popular clustering techniques, and it has been adapted to many problem domains because of its simplicity and efficiency. This algorithm was voted as one of the top 10 algorithms in the data mining research area for identifying hidden patterns and revealing underlying knowledge from large data collections [17].

After calculating the RRI differences for each time series, the Simple K-Means algorithm is used to cluster the RRI differences. In this algorithm, parameter *k* represents the number of clusters desired. The output of the clustering algorithm is *k* clusters, which should correspond to any known classes in terms of instance distribution.

**Figure 4:**
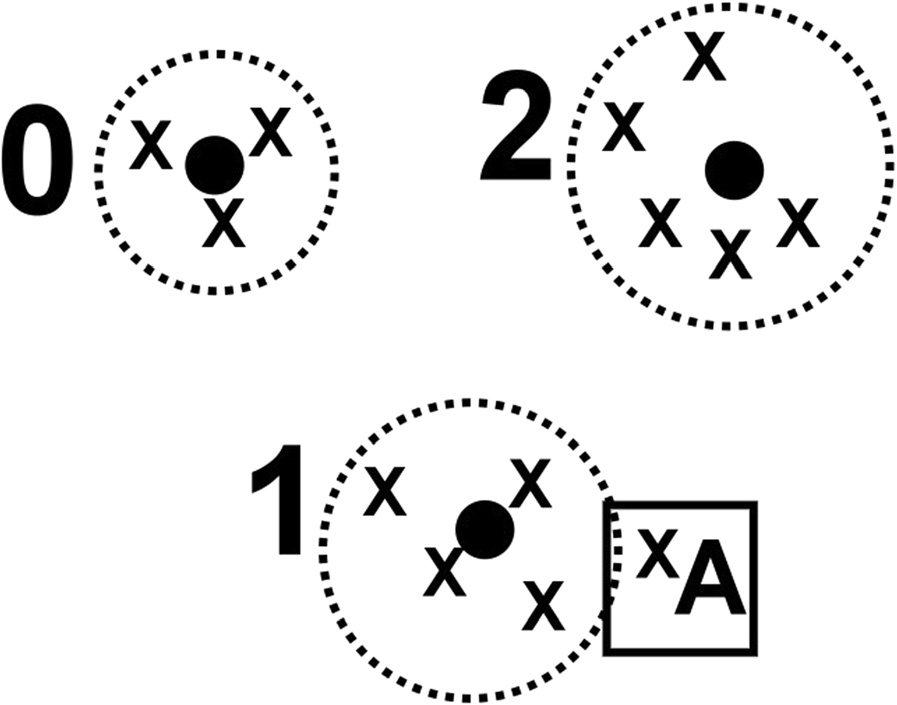
**An example to demonstrate the Simple K-Means algorithm with 13 data points when *k *= 3.**

Figure 4 is a demonstration of the Simple K-Means algorithm with 13 data points when *k* = 3. The coordinates of the black points are the mean values of the coordinates of the points of the cluster. In this example, the distance between the centroid of the cluster 1 and the point A was smallest, and therefore point A will be assigned to cluster 1.

**Figure 5:**
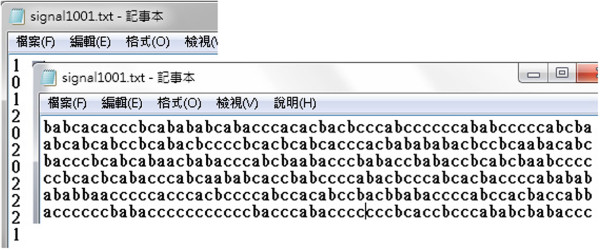
Example after being mapped when the number of cluster is 3.

In the AIIA method, every RRI difference can be assigned to a cluster number. In this paper, *k* = 2 to 26 were tested. Each cluster number is then mapped to one of the 26 English letters. Figure 5 is an example of the symbolization of a sample when *k* = 3.

### Identifying styles and signatures – Using the n-gram algorithm

**Figure 6:**
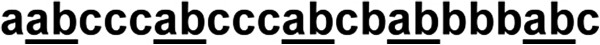
Example to generate each 2-gram “ab”.

An n-gram distribution computes the number of occurrence of each “gram”. Figure 6 displays an example to generate each 2-gram “ab”. Each 2-gram is displayed by the underline. The occurrence of 2-gram “ab” is 5. Note that strings “ab” and “ba” have exactly the same two letters “a” and “b”, but the two strings “ab” and “ba” are clearly not the same.

This research uses 26 clusters (*k* = 26) with 1-gram, 2-gram and 3-gram for analysis, which includes 18,278 (18,278 = 26^1^ + 26^2^ + 26^3^) different kinds of string combinations. That is to say, 18,278 different kinds of variations in the sample are considered.

### Classification – Using Classic classifiers

Prior to classification, a probability matrix according to the occurrences of each gram in the last step is generated. Then 6 classic classifiers, including Bayesian Network, Logistic, Naïve Bayesian, Neural Network, Support Vector Matrix (SVM) and Tree-J48, are used to classify the samples into different heart diseases.

In the next section, two examples are used to demonstrate how the AIIA method is applied to categorize different types of heart rate time series. The databases for the examples were provided by PhysioBank, which was created under the auspices of the National Center for Research Resources of the National Institutes of Health, USA. It is a large and growing archive of well-characterized digital recordings of physiological signals and related data for use by the biomedical research community. The biomedical signals from healthy subjects and from patients with a variety of diseases are included [18].

The 10-fold cross-validation is used to assess the result. In the 10-fold cross-validation, the original samples were randomly partitioned into 10 subsets. Of the 10 subsets, a single subset was retained as the validation data for testing the model and the remaining 9 subsets were used as training data. This step was then repeated 10 times. Each subset was used exactly once as the validation data. Finally, the 10 results from the 10 subsets were averaged to produce a single estimation. The advantage of this method was that all observations were used for both training and validation and each observation was used for validation exactly once.

## Results

### Example 1 – Using the AIIA method to classify heart diseases

In this first example, the AIIA method is used to classify heart diseases from the heart rate time series. There are 142 samples of heart rate time series data in this example, which can be divided into 5 groups, including 43 samples with Congestive Heart Failure (CHF), 9 samples with Atrial Fibrillation (AF), 20 samples of healthy young subjects (HY), 20 samples of healthy elderly subjects (HE), and 50 samples of white noise (WNU). Table 1 presents detailed information on the 5 groups.

**Table 1:** The 5 groups of the example 1

**No.**	**Group**	**Subjects**	**Description**	**Source**
1.	Congestive Heart Failure (CHF)	43	15 females and 28 males, average age 55.5 years. It takes 16 to 24 hours for each sample (around 75,000 RRI).	BIDMC Congestive Heart Failure Database [19]
2.	Atrial Fibrillation (AF)	9	Takes only 2 hours for recording (around 12,000 RRI).	Albert C.-C. Yang
3.	Healthy Young (HY)	20	10 females and 10 males, average age 25.9 years. It takes 2 hours for each sample (around 7,100 RRI).	Fantasia Database [20,21]
4.	Healthy Elderly (HE)	20	10 females and 10 males, average age 74.5 years. It takes 2 hours for each sample (around 7,200 RRI).	
5.	White Noise (WNU)	50	Uniform distribution. It takes 6 hours for each sample (around 15,000 RRI).	Artificially generated
	*Total*	**142**		

The AIIA method is first used to generate the symbolic sequences of each sample, to identify styles and signatures, and to calculate the probability of each signature. Then 6 classic classifiers were used to classify the 142 samples into 5 groups, AF, CHF, HY, HE, and WNU using the probability of each signature. Table 2 shows the top 4 classified results by using 2-gram analysis.

**Table 2:** The top 4 classified results (2 gram, 26 clusters)

**Classifier**	**Cluster Number (*k*)**	**Total number of instances**	**Correctly classified instances**	**Incorrectly classified instances**	**Accuracy**	**Best Performance**
Bayesian Network	20	142	126	16	88.7%	20 clusters, 88.7%
SVM	20	142	124	18	87.3%	24 clusters, 88.7%
Tree-J48	20	142	121	21	85.2%	24 clusters, 88.0%
Naïve Bayse	20	142	113	29	79.6%	24 clusters, 81.7%

Accuracy at *k* = 20 and the best accuracy of each classifier are presented for comparison.

**Figure 7:**
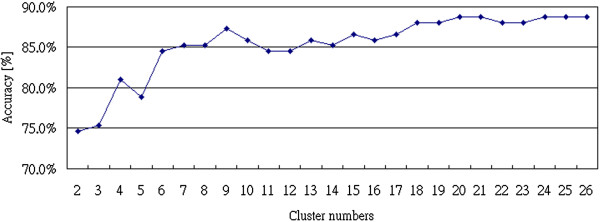
Relationship between the number of clusters and accuracy rate of 2-gram analysis.

From the results in Table 2, the Bayesian Network had the best performance with 88.7% accuracy in classifying the samples from patients with different heart diseases when the cluster number *k* = 20. The Support Vector Matrix (SVM) and the Tree-J48 also had over 88.0% accuracy, but both of them needed 24 clusters. On the other hand, the classification results using the other classifiers were unstable. Figure 7 shows the relationship between accuracy rates and cluster numbers by using the Bayesian Network for classification. When the cluster number was over 16, the performance of the classifier became stable.

Table 3 shows details for the classification results by using the Bayesian Network. The Bayesian Network has 77.8% accuracy for classifying the AF, 88.4% accuracy for classifying the CHF, 85.0% accuracy for classifying the HE, 65.0% accuracy for classifying the HY, and 100% accuracy for classifying the WNU. The best performance was in classifying the CHF group, which had profoundly abnormal heart function. This function was associated with pathological alterations in both the parasympathetic and sympathetic control mechanisms.

**Table 3:** Detailed classification results form using Bayesian Network

**Group**	**AF**	**CHF**	**HE**	**HY**	**WNU**
Total	9	43	20	20	50
Correct	7	38	17	13	50
Incorrect	2	5	3	7	0
Accuracy	77.8%	88.4%	85.0%	65.0%	100%

**Figure 8:**
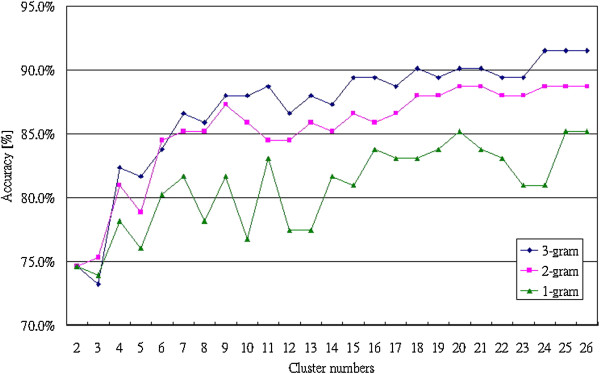
The comparison result between 1-gram, 2-gram and 3-gram by using Bayesian Network.

Figure 8 shows the comparison between the results from 1-gram, 2-gram and 3-gram by using the Bayesian Network. When cluster numbers are more than 7, the accuracies by using 3-gram analysis are better than the classified results by using 1-gram and 2-gram analysis. AIIA method achieved 91% (3-gram, 26 clusters) accuracy in successfully classifying between the patients with Atrial Fibrillation (AF), Congestive Heart Failure (CHF) and healthy people. The same sample data was also studied by [10]. However, no accuracy data was presented and therefore cannot be compared.

### Example 2 – Using the AIIA method to classify patients with apnea

Apnea is a term for breathing suspension. There is no movement of patient’s muscles of respiration and it leads to lack of oxygen in the blood circulation. Thus, patients with sleep apnea may have an increased cardiac risk [22]. In the second example, the AIIA method is used to classify the patients with apnea from the heart rate time series. There are 4 groups in this example, including 20 samples with Apnea, 20 samples of healthy young subjects (HY), 20 samples of healthy elderly subjects (HE), and 20 samples of white noise (WNU) for a total of 80 samples. Table 4 presents detailed information on the 4 groups.

**Table 4:** The 4 groups of the example 2

**No.**	**Group**	**Subject**	**Description**	**Source**
1.	Apnea (APNEA)	20	This experiment uses the class ‘A’ set which includes 20 records for the target set of Apnea. These records meet all Apnea criteria. Recordings in class A contain at least one hour with an apnea index of 10 or more, and at least 100 minutes with apnea during the recording. It takes 8 hours for each sample (around 35,000 RRI).	Apnea-ECG database [23]
2.	Health Young (HY)	20	10 females and 10 males, average age 55.5 years. It takes 2 hours for each sample (around 7,100 RRI).	Fantasia Database [20,21]
3.	Health Elderly (HE)	20	10 females and 10 males, average age 74.5 years. It takes 2 hours for each sample (around 7,200 RRI).	
4.	White Noise (WNU)	20	Uniform distribution. It takes 6 hours for each sample (around 15,000 RRI).	Artificially generated
	*Total*	**80**		

The AIIA method is first used to generate the symbolic sequences of each sample, identify styles and signatures, and calculate the probability of each signature. Then 6 classic classifiers were used to classify the 80 samples into 4 groups, APNEA, HY, HE, and WNU using the probability of each signature. Table 5 shows the top 4 classified results by using the 2-gram analysis.

**Table 5:** Top 4 classification results (2 gram, 26 clusters)

**Classifier**	**Cluster Number (*k*)**	**Total number of instances**	**Correctly classified instances**	**Incorrectly classified instances**	**Accuracy**	**Best Performance**
Bayesian Network	11	80	68	12	85.0%	11 clusters, 85.0%
Tree-J48	11	80	63	17	78.6%	17 clusters, 81.3%
Logistic	11	80	55	25	68.8%	17 clusters, 83.8%
SVM	11	80	54	26	67.5%	23 clusters, 83.8%

Accuracy at *k* = 11 and the best accuracy of each classifier are presented for comparison

**Figure 9:**
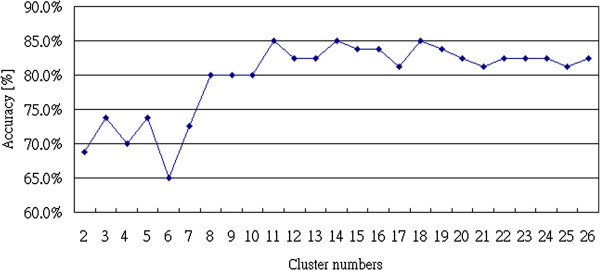
Relationship between the number of clusters and accuracy rate of 2-gram analysis.

From the results in Table 5, the Bayesian Network again had the best performance with 85.0% accuracy in classifying the samples from patients with different heart diseases when the cluster number *k* = 11. The Logistic method and the SVM had 83.8% accuracy in classifying the data. Figure 9 shows the relationship between accuracy rates and cluster numbers by using the Bayesian Network for classification. From Figure 9, there is a big difference between clusters 5 to 7, and when the cluster number was over 16, the performance of the classifier became stable.

Table 6 describes the detailed classification results using the Bayesian Network. The Bayesian Network provides 95% accuracy in classifying the Apnea, 85% accuracy in classifying the HE, 60% accuracy in classifying the HY, and 100% accuracy in classifying the WNU. Its best performance was classifying the Apnea group.

**Table 6:** Detailed classification results using Bayesian Network

**Group**	**Apnea**	**HE**	**HY**	**WNU**
Total	20	20	20	20
Correct	19	17	12	20
Incorrect	1	3	8	0
Accuracy	95.0%	85.0%	60.0%	100%

**Figure 10:**
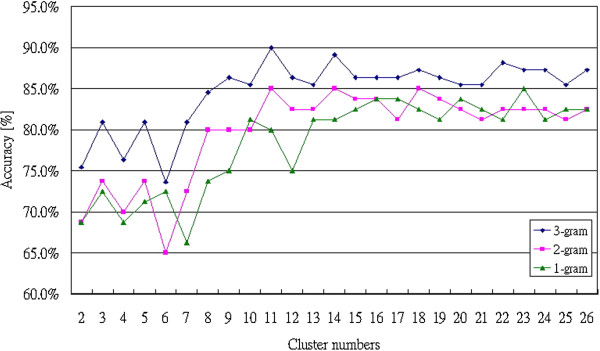
The comparison result between1-gram, 2-gram and 3-gram by using Bayesian Network.

Figure 10 shows the comparison results between 1-gram, 2-gram and 3-gram by using the Bayesian Network. Obviously, the classification results by using 3-gram analysis are better than those by using 1-gram and 2-gram analysis because more variations are considered in 3-gram analysis. The AIIA method achieved 87% (3-gram, 26 clusters) accuracy in classifying the patients with apnea.

## Discussion

As interest continues to grow in analyzing heart diseases, symbolic analysis will clearly remain an important research tool. It offers advantages such as computational efficiency, ease of visualization, as well as the ability to combine with other algorithms, information theories and language that may not be matched by any other approach. The most significant issue in the application of symbolic analysis is how to develop an algorithm to appropriately define symbols in the absence of generating partitions. Although some information is always lost during the symbolic transformation process and it involves some degree of imprecision, many associated applications have proved it to be viable and realistic.

The AIIA method presented here also cannot assure that no information is lost, but it tries to capture small variations when doing the symbolic transformation. First, the method uses up to 26 symbols (a to z) to represent variations between interbeat intervals to show the increase or decrease phases and the degree of variation. Second, the symbols are not generated by artificial experiences or functions, but by the Simple K-Means algorithm, which is one of the most popular clustering techniques that supplies clusters with minimal total variance [17]. The criterion of minimal total variance yields the most closed clusters. That is, if variations belong to the same cluster, they are similar. This step is totally different from previous studies. Finally, it uses the n-gram algorithm to generate symbolic sequences. Closely associated with the problem of symbol definition, there always needs to be an efficient algorithm for defining the appropriate length of symbolic sequences. The n-gram algorithm can automatically change the lengths of sequences according to the experimental performance. The complexity of calculating the occurrence of each “gram” is, where n is the number of clusters and m is the number of grams. In general, more clusters and grams may lead to better performance, but it requires a large amount of computation and takes a long CPU time. It also may lead to an overfitting problem.

## Conclusions

Biological signals may carry specific characteristics that reflect basic dynamics of the body. Therefore, finding and analyzing the hidden signals of dynamical structures which raise a lot of clinical interests. The AIIA method presented here uses the Simple K-Means algorithm for symbolization, which offers a new way to represent subtle variations between two interbeat intervals without human intervention.

The two experiments presented in this paper demonstrate that AIIA method can categorize different heart diseases. Both experiments acquired the best category results when using the Bayesian Network. For future work, the concept of the AIIA method can be extended to the categorization of other physiological signals. Further study is required to show robustness of the AIAA method, and more features can be added to improve its accuracy.

## Competing interests

The authors have no competing interests.

## Authors’ contributions

YCH, HL and JLL conceived the study. YCH and HL participated in the acquisition of data, designed the experiment, wrote the program, and drafted the manuscript. YLH revised and restructured the study and the manuscript. All of authors read and approved the final manuscript.

## Pre-publication history

The pre-publication history for this paper can be accessed here:

http://www.biomedcentral.com/1472-6947/12/64/prepub
